# Cystathionine Levels in Patients With Huntington Disease

**DOI:** 10.1371/currents.hd.c63b441d04bb6738c0234f91c2b3e312

**Published:** 2015-09-16

**Authors:** N.A. Aziz, W. Onkenhout, H.J. Kerstens, Raymund A.C. Roos

**Affiliations:** Department of Neurology, Leiden University Medical Centre, Leiden, the Netherlands; Department of Clinical Chemistry and Laboratory Medicine, Unit of Metabolic Diseases, Leiden University Medical Centre, The Netherlands; Department of Clinical Chemistry and Laboratory Medicine, Unit of Metabolic Diseases, Leiden University Medical Centre, The Netherlands; Department of Neurology, Leiden University Medical Centre, Leiden, the Netherlands.

## Abstract

Background: Recently a profound depletion of cystathionine γ-lyase (CSE), the principal enzyme involved in the generation of cysteine from cystathionine, was shown in Huntington disease (HD) patients and several transgenic HD mouse models. We therefore hypothesized that blood and urine cystathionine levels may be increased in HD patients and that this increase might correlate with disease progression. Methods: We measured concentrations of cystathionine as well as 22 other amino acids in fasting plasma and 24-h urine samples of nine early-stage HD patients and nine age, sex, and body mass index matched controls. Results: There were no significant differences in the plasma or urine concentrations of cystathionine or any other amino acid between HD patients and controls. Conclusion: We found no evidence for changes in plasma or urine concentrations of cystathionine in early-stage HD patients. Therefore, cystathionine levels are unlikely to be useful as a state biomarker in HD.

## Introduction

Huntington disease (HD) is an autosomal dominant, neurodegenerative disorder caused by an expanded trinucleotide (CAG) repeat sequence in the first exon of the HD (*HTT*) gene, leading to an enlarged polyglutamine tract in the encoded protein huntingtin.^1^ Unwanted choreiform movements, psychiatric and behavioural disturbances and cognitive impairment characterize the disease. Other less well-known, but debilitating manifestations of HD include weight loss, sleep disturbances and autonomic nervous system (ANS) dysfunction.^2^ Unfortunately, there are no disease-modifying therapies available, although a number of potential disease-modifying drugs are currently in development.^2^ In order to rapidly assess these drugs in clinical trials there is a pressing need for reliable biomarkers with a high sensitivity to disease progression.^3^ As HD is a slowly progressive disease such biomarkers could initially be applied as surrogate trial end-points to allow rapid prioritization of potentially effective drugs. Subsequently, promising candidate drugs could be tested further for clinical efficacy in randomized trials using suitable clinical end-points.^3^

Recently a substantial depletion of cystathionine γ-lyase (CSE), the principal enzyme involved in the generation of cysteine from cystathionine, was shown in HD.^4^^,^ 5 The levels of this enzyme were profoundly decreased in HD striatal cell lines containing 111 glutamine residues, in brains of two transgenic HD mouse models (i.e. R6/2 and Q175 mice) as well as in post-mortem brain samples of HD patients.^4^ Importantly, the levels of CSE in liver and pancreatic lysates of R6/2 mice were decreased to a similar extent as those in the brain, purportedly due to an aberrant interaction of the mutant huntingtin protein with specificity protein-1, a transcriptional activator of CSE.^4^ Paralleling findings in individuals with inactivating mutations of the cystathionine γ-lyase (CTH) gene which encodes CSE, a depletion of CSE might result in elevated levels of cystathionine in both blood and urine.^6^ Therefore, we hypothesized that the levels of cystathionine in both blood and urine may be increased in patients with HD compared to matched controls and that this increase might correlate with disease progression.

## Methods

*Clinical protocol*

We used data and plasma/urine samples collected in our earlier studies, the protocols of which have been described previously.^7^^,^ 8 In brief, nine early-stage HD patients and nine healthy control subjects, matched for age, sex, and body mass index (BMI), were enrolled.^7^^,^ 8 In the patient group, mutant CAG repeat size ranged between 41 and 50. The clinical diagnosis of HD was made by a neurologist specialized in movement disorders (R.A.C.R.). The Unified Huntington Disease Rating Scale (UHDRS) was used to assess HD symptoms and signs. All subjects were free of medication. Subjects were eligible for participation after exclusion of hypertension, any known (history of) pituitary disease, recent intentional weight change (>3 kg weight gain or loss within the last 3 months), and any other chronic conditions except HD. Written informed consent was obtained from all subjects. The study was approved by the ethics committee of the Leiden University Medical Centre. Subjects were admitted to the Clinical Research Center for blood sampling. A cannula was inserted into an antecubital vein and 2-3 mL blood samples were collected with S-monovetten (Sarstedt, Etten-Leur, The Netherlands). Sampling started at 16:30 and continued for 24 hours at 10-min intervals. EDTA tubes were put immediately on ice and centrifuged within an hour at 1610 g at 4 ºC for 20 min, and plasma was stored at -80 ºC. Three standardized meals were served at 09:00, 13:00, and 19:00 h (Nutridrink, 1.5 kcal/ml, 1500–1800 kcal/d; macronutrient composition per 100 ml: protein, 5 g; fat, 6.5 g; carbohydrate, 17.9 g; Nutricia, Zoetermeer, The Netherlands). Subjects remained sedentary except for bathroom visits. Furthermore, twenty-four hour urine was collected and stored at -80 ºC. No daytime naps were allowed. Lights were switched off at 23:00 h and, the next morning, subjects were awakened at 07:30 h. Bioelectrical impedance analysis was used to assess lean body mass and fat percentage at 08:00 h.

*Assays*

Concentrations of cystathionine as well as 22 other amino acids (alanine, arginine, asparagine, aspartic acid, citrulline, glutamine, glutamic acid, glycine, histidine, isoleucine, leucine, lysine, methionine, ornithine, phenylalanine, proline, serine, taurine, threonine, tryptophan, tyrosine and valine) were determined in fasting plasma samples obtained at 08:30 h and samples from 24-hour urine. Additionally, cystine levels were determined in urine samples only as the levels of this amino acid could not be reliably quantified in stored plasma samples. All samples were analysed once according to a procedure described before^9^ with minor modifications, on a Biochrom 30 automated amino acid analyser (Biochrom, Cambridge, UK) using standard conditions for physiological amino acid separation. The minor modifications were: 250 µL of plasma (and urine) were used instead of 400 µL, 40 µL of the plasma supernatant (and 20 µL of the urine supernatant) was injected instead of 60 µL and a 13 mm membrane filter was used instead of a 25 mm membrane filter. The detection limits of the assays were 1 μmol/L for plasma and 1 μmol/mmol creatinine for urine and total analysis time was 173 min.

*Statistical analysis*

Results are presented as medians and interquartile ranges unless otherwise specified. Because of small group sizes the non-parametric Mann-Whitney *U*-test was applied to assess intergroup differences, while Spearman’s correlation coefficient was used to assess all correlations. All tests were two-tailed and significance level was set at p < 0.05.

## Results

*Subjects*

The HD and the control group did not differ with respect to age, sex, body mass index, body fat or lean body mass (all p ≥ 0.27 , **Table 1**).

**Table 1:** Characteristics of the study population

	HD patients*	Controls*	p-value
Male/female	6/3	6/3	-
Age [y]	47.1 (3.4)	48.6 (3.3)	0.691
BMI	24.1 (1.0)	24.3 (0.6)	0.691
Fat [%]	25.5 (2.4)	25.6 (2.4)	0.825
Lean body mass [kg]	57.3 (3.2)	56.2 (3.0)	0.691
Waist-to-hip ratio	0.89 (0.03)	0.94 (0.02)	0.270
Mutant CAG repeat size	44.4 (1.0)	-	-
Age of onset [y]	41.4 (3.0)	-	-
Disease duration [y]	5.7 (1.1)	-	-
UHDRS motor score	22.2 (6.0)	-	-
TFC score	11.7 (0.7)	-	-

*) Values are indicated as mean (SE).

Abbreviations: BMI = Body Mass Index; TFC = Total Functional Capacity; UHDRS = Unified Huntington’s Disease Rating Scale.

*Amino acid concentrations*

There were no significant differences in plasma or urine concentrations of cystathionine between HD patients and controls (all p ≥ 0.102, **Table 2**). Neither did the levels of the other amino acids differ between HD patients and controls (all p ≥ 0.102, **Table 2**).

**Table 2:** Amino acid concentrations

	Plasma^a^		Urine^b^	
	HD patients	Controls	HD patients	Controls
Alanine	256 (235-393)	288 (236-444)	37.0 (21.5-43.2)	28.5 (27.6-39.1)
Arginine	99 (69-107)	102 (71-109)	2.2 (1.5-2.7)	2.0 (1.7-2.9)
Asparagine	42 (34-51)	52 (37-58)	13.6 (8.8-23.2)	14.8 (10.7-16.0)
Aspartic acid	9 (8-13)	11 (11-13)	12.6 (11.3-14.2)	11.6 (10.7-12.5)
Citrulline	38 (32-47)	42 (39-45)	0.5 (0.5-2.0)^c^	0.5 (0.5-0.8)^c^
Cystathionine	2 (2-3)	2 (2-3)	2.3 (0.9-2.7)^c^	1.1 (0.5-2.5)^c^
Cystine	-	-	7.1 (5.8-9.7)	5.6 (4.8-6.5)
Glutamine	626 (530-685)	611 (550-692)	59.3 (35.9-84.0)	45.3 (43.0-57.8)
Glutamic acid	40 (32-67)	40 (34-52)	2.9 (2.3-4.0)	2.7 (2.0-3.3)
Glycine	223 (158-282)	256 (209-307)	129.1 (105.2-286.0)	136.1 (118.5-197.7)
Histidine	75 (63-92)	82 (74-88)	78.1 (46.6-139.7)	79.3 (59.0-94.7)
Isoleucine	60 (53-80)	68 (57-82)	1.5 (0.5-1.7)^c^	1.1 (0.5-1.6)^c^
Leucine	133 (104-149)	140 (101-164)	2.9 (2.0-4.1)	3.0 (2.2-3.4)
Lysine	183 (141-218)	187 (176-213)	24.6 (20.9-49.0)	23.4 (20.6-30.9)
Methionine	24 (20-32)	24 (22-34)	2.3 (1.7-2.5)	1.8 (1.4-2.4)
Ornithine	47 (42-58)	47 (39-59)	2.6 (1.3-3.1)	3.0 (1.7-3.9)
Phenylalanine	62 (51-76)	61 (53-73)	6.8 (5.3-8.7)	5.6 (4.1-6.7)
Proline	191 (154-247)	190 (168-264)	ND	ND
Serine	105 (77-125)	111 (107-122)	43.8 (24.0-65.0)	41.4 (35.9-44.6)
Taurine	33 (29-43)	34 (32-43)	84.2 (33.5-100.7)	64.3 (22.9-95.9)
Threonine	113 (95-161)	123 (113-146)	19.1 (10.9-25.6)	15.9 (11.2-18.1)
Tryptophan	59 (36-72)	56 (50-67)	ND	ND
Tyrosine	55 (42-64)	55 (49-66)	10.1 (7.3-16.8)	9.8(6.6-12.1)
Valine	246 (202-257)	247 (203-261)	4.4 (3.2-6.9)	3.9 (3.3-5.0)

Results are presented as medians (interquartile range). ND: not detectable. There were no significant intergroup differences for any amino acid either in plasma or in urine (all p ≥ 0.102).

^a^) Plasma concentrations are in μmol/L.

^b^) Urine concentrations are in μmol/mmol creatinine.

^c^) In some participants amino acid concentrations were below the limit of detection of the assay. In order to calculate summary measures, the expected amino acid concentrations in these subjects were assumed to be half of the detection limit.

*Association with clinical features*

In HD patients plasma cystathionine levels did not correlate with any UHDRS domain score (all p ≥ 0.33). Conversely, urine cystathionine levels were significantly correlated with the total functional capacity score (r = -0.75, p = 0.020), but not with total motor score (r = +0.44, p = 0.242). Urine cystine levels were associated with both total motor score (r = +0.67, p = 0.050) and total functional capacity score (r = -0.71, p = 0.032). There were no significant associations between cystathionine levels in either plasma or urine and total behavioural score, CAG repeat size or body mass index (all p ≥ 0.71). Of the other amino acids studied urine levels of arginine, aspartic acid, citrulline, isoleucine, leucine and taurine were significantly associated with either total motor score or total functional capacity score (all p < 0.050). Plasma levels of none of these and other amino acids were associated with either total motor score or total functional capacity (all p ≥ 0.092).

## Discussion

As recently reported^4^ ^5^, a major decrease of CSE, which is the main generator of cysteine from cystathionine, would be expected to result in increased levels of cystathionine in HD patients. However, in this pilot study we found similar concentrations of cystathionine in both fasting plasma samples and samples from 24-hours urine in early-stage HD patients and matched controls. There are several likely explanations for this apparent discrepancy. First, although Paul et. al demonstrated significantly decreased levels of CSE in brains of HD patients, they did not study levels of CSE in peripheral tissues of these patients. Moreover, the depletion of CSE levels in hepatic and pancreatic tissue of R6/2 mice was less pronounced than in their brain tissue.^4^ Therefore, it could be that CSE depletion only leads to detectable changes in cystathionine levels in brain and/or cerebrospinal fluid, but not peripheral tissues, of HD patients. Second, the post-mortem patient material studied by Paul et al. likely originated from end-stage HD patients, whereas we studied early-stage patients. Thus, given the significant associations between cystathionine levels and disease severity in our small group of patients, it is conceivable that in more advanced HD patients levels of cystathionine will indeed become abnormal.

We neither found any evidence for decreased levels of other amino acids including alanine or the branched chain amino acids isoleucine, leucine and valine in HD patients as reported earlier.^11^^,^ 12 ^,^ 13 We did find an association between urine levels of, among others, isoleucine and leucine and disease severity in our group of early-stage HD patients suggesting that the levels of some amino acids might become abnormal with disease progression, although these associations should be interpreted cautiously given the low amino acid concentrations. Another possible explanation for the lack of differences in amino acid levels between our patient and control group could be that in contrast to previous studies we also accounted for dietary intake by providing the same standardized meals to all participants thereby decreasing confounding effects mediated through differences in dietary composition.^10^ However, in any case our study does not suggest large changes in any amino acid in early-stage HD patients.

In conclusion, we found no evidence for changes in plasma or urine concentrations of cystathionine or any other amino acid in early-stage HD patients. Although we found associations between cystathionine, as well as several other amino acids, and disease severity, the potential of these amino acids to serve as state biomarkers in HD needs further validation in larger groups of patients.

## Competing Interest statement

The authors have declared that no competing interests exist.
